# Development of an Adeno-Associated Virus-Vectored SARS-CoV-2 Vaccine and Its Immunogenicity in Mice

**DOI:** 10.3389/fcimb.2022.802147

**Published:** 2022-03-03

**Authors:** Xi Qin, Shanhu Li, Xiang Li, Dening Pei, Yu Liu, Youxue Ding, Lan Liu, Hua Bi, Xinchang Shi, Ying Guo, Enyue Fang, Fang Huang, Lei Yu, Liuqiang Zhu, Yifang An, C. Alexander Valencia, Yuhua Li, Biao Dong, Yong Zhou

**Affiliations:** ^1^ Department of Recombinant Products, National Institutes for Food and Drug Control, Beijing, China; ^2^ Department of Cell Engineering, Beijing Institute of Biotechnology, Beijing, China; ^3^ Department of Geriatrics and National Clinical Research Center for Geriatrics, State Key Laboratory of Biotherapy, West China Hospital, Sichuan University, Chengdu, China; ^4^ Department of Arboviral Vaccine, National Institutes for Food and Drug Control, Beijing, China

**Keywords:** SARS-CoV-2, AAV9, vaccine, immune, long term protection

## Abstract

Owing to the outbreak of the novel coronavirus (SARS-CoV-2) worldwide at the end of 2019, the development of a SARS-CoV-2 vaccine became an urgent need. In this study, we developed a type 9 adeno-associated virus vectored vaccine candidate expressing a dimeric receptor binding domain (RBD) of the SARS-CoV-2 spike protein (S protein) and evaluated its immunogenicity in a murine model. The vaccine candidate, named AAV9-RBD virus, was constructed by inserting a signal peptide to the N-terminus of two copies of RBD, spaced by a linker, into the genome of a type 9 adeno-associated virus. *In vitro* assays showed that HeLa cells infected by the recombinant AAV virus expressed high levels of the recombinant RBD protein, mostly found in the cell culture supernatant. The recombinant AAV9-RBD virus was cultured and purified. The genome titer of the purified recombinant AAV9-RBD virus was determined to be 2.4 × 10^13^ genome copies/mL (GC/mL) by Q-PCR. Balb/c mice were immunized with the virus by intramuscular injection or nasal drip administration. Eight weeks after immunization, neutralizing antibodies against the new coronavirus pseudovirus were detected in the sera of all mice; the mean neutralizing antibody EC_50_ values were 517.7 ± 292.1 (n=10) and 682.8 ± 454.0 (n=10) in the intramuscular injection group and nasal drip group, respectively. The results of this study showed that the recombinant AAV9-RBD virus may be used for the development of a SARS-CoV-2 vaccine.

## Introduction

At the end of 2019, there was an outbreak of the novel coronavirus (SARS-CoV-2), leading to an ongoing pandemic that has caused five million deaths, since November of 2011, and two hundred million cases. The SARS-CoV-2 virus is a positive-strand RNA virus belonging to the coronavirus family that had never been reported in humans previously (Zhu et al., 2020). After infecting a human body, SARS-CoV-2 can cause severe inflammation in the lungs and even death. Studies have shown that SARS-CoV-2 binds to human cell receptor angiotensin-converting enzyme 2 (ACE2) through the receptor-binding domain (RBD) of the spike glycoprotein, thus triggering a viral invasion of the host cells (Zhou et al., 2020). At present, RBD has become an important target for the development of therapeutic drugs and vaccines against SARS-CoV-2 (Wu et al., 2020). The RBD-based protein subunit vaccine (Min and Sun, 2021; Yang et al., 2021) and mRNA vaccine (Tai et al., 2020; Karpenko et al., 2021) have achieved good clinical results, exhibiting excellent immunogenicity in animal models and humans. At least one such vaccine is authorized for emergency use (Yang et al., 2021). Several mRNA vaccines or adenovirus vectored vaccines-based on the full length S protein are also licensed for use as this article is being written.

The duration of protection conferred by a vaccine is an important indicator of the vaccine’s effectiveness. Studies have shown that neutralizing antibodies in SARS-CoV-2 patients declined rapidly after recovery (Long et al., 2020). The immunity generated by all currently licensed vaccines decreases within months and has raised the need for booster immunization in many countries. Therefore, the development of a vaccine that can continuously stimulate the body to produce high levels of neutralizing antibody over long period of time represents an improvement to the current vaccines.

The wild-type adeno-associated virus is a single stranded, linear, DNA deficient virus that has never been found to cause any human disease (Schultz and Chamberlain, 2008; Hüser et al., 2010). Recombinant adeno-associated virus (rAAV) is derived from non-pathogenic wild-type AAV, in which *rep*, which is related to replication, and *cap*, which encodes the shell protein, are replaced by foreign genes. After inoculation into a human body, recombinant adeno-associated viruses carrying the exogenous gene can continuously express the antigen in the human cells for more than 3 years (Pasi et al., 2020) and even up to 15 years (George et al., 2020). In 2012, the EMA (European Medicines Agency) approved Glybera (rAAV1 as the vector) for the treatment of lipoprotein lipase deficiency. In 2017, the FDA (Food and Drug Administration) unanimously approved Luxturna (rAAV2 as the vector) for the treatment of Leber congenital amaurosis. In 2019, the FDA approved Zolgensma (rAAV9 as the vector) for the treatment of spinal muscular atrophy in children. As of November 2018, a total of 145 clinical trials involving rAAV were registered in *ClinicalTrials.gov* (Wang et al., 2019). Because of its advantages, including low immunogenicity and cytotoxicity, wide host range, stable physical and chemical properties, and ability to express exogenous genes over the long-term (van der Laan et al., 2011; Dismuke et al., 2013; Wang et al., 2019), rAAV is considered as a safe and effective virus vector (Schultz et al., 2008; Donsante et al., 2007). It has been widely used in clinical trials for gene therapy (Rakoczy et al., 2015; Bennett et al., 2016; George et al., 2017; Mueller et al., 2017; Rangarajan et al., 2017) and the recombinant adeno-associated virus may be also effectively used for vaccine development (Demminger et al., 2020).

In this study, we selected the recombinant adeno-associated virus type 9 with a relatively wide tissue tropism (Zincarelli et al., 2008) as a vector to develop an RBD-based SARS-CoV-2 vaccine. Recombinant RBD protein is expressed and secreted by the infected cells over a long period of time. After mice were immunized with this virus, neutralizing antibodies against the SARS-CoV-2 pseudovirus persisted and may offer durable protection.

## Materials and Methods

### Reagents and Materials

HEK293 cells and HeLa cells were purchased from ATCC, cultured in DMEM containing 10% fetal bovine serum and 1% Penicillin-Streptomycin. All culture reagents were purchased from GIBCO (USA). The inserted gene was synthesized by Synbio Technologies Co., Ltd. (China). pAAV-MCS, pAAV-RC9, and pAAV-Help were purchased from Biofeng (China). The transfection reagent Lipofectamine 3000 was purchased from Thermo Fisher (USA). Three plasmid transfection reagents, FectoVIR-AAV, were purchased from Polyplus (USA). The anti-RBD monoclonal antibody was obtained from Sino Biological (China). The plasmid extraction kit was purchased from QIAGEN (USA). The AAVpro Titration Kit (for Real Time PCR) Ver.2 was obtained from TaKaRa (Japan). The benzonase and iodixanol reagents were purchased from Sigma (USA). The luciferase detection reagent was obtained from PerkinElmer (USA). The novel corona pseudovirus and Huh-7 cells were donated by Professor Wang Youchun and Professor Huang Weijin, respectively, from the National Institutes for Food and Drug Control.

### Construction of the Eukaryotic Expression Vector

**Figure 1:**
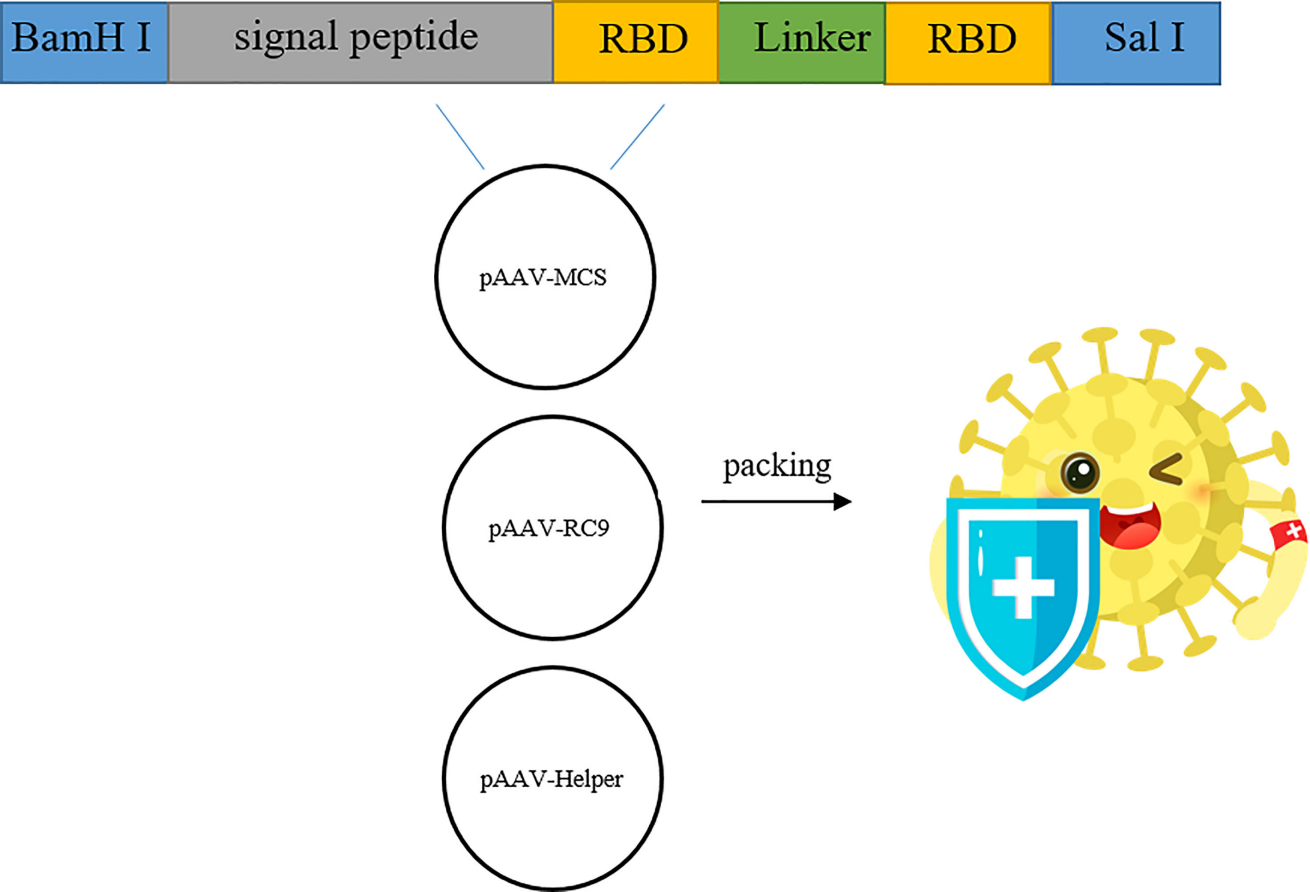
Construction and genome composition of recombinant AAV9-RBD. Four pAAV-RBD plasmids were constructed. Clone 1 contains tPA signal peptide in self-complementary form. Clone 2: contains tPA signal peptide in single-stranded form. Clone 3 contains Glu signal peptide in self-complementary form. Clone 4 contains Glu signal peptide in single-stranded form.

After the synthetic target gene (**Figure 1**) and pAAV-MCS vector were digested with BamH I and Sal I, respectively, the target gene and the vector were ligated and used to transform *E. coli* DH5α competent cells. Transformed cells were plated on LB culture medium plates with kanamycin. Positive clones were screened, identified, and sequenced. The sequence of RBD used was at amino acids 319 to 541 of the Spike protein (WH-Human_1). The signal peptide tPA had the specific sequence of MDAMKRGLCCVLLLCGAVFVSA, while Glu had the sequence MGVKVLFALICIAVAEVTG, and the sequence of GC linker was GSGGSG.

### Transfection

HEK293 cells were seeded onto a 24-well plate at a density of 40,000-50,000 cells/well, and the culture medium was replaced on the second day of inoculation. After 2 h, the recombinant expression plasmid was used to transfect cells using Lipofectamine 3000 at a ratio of 0.5 μg/well. Cells were incubated at 37°C with 5% CO2 for 6 h, and subsequently cultured in new media for 48 h. At the end, cell lysates and cell culture supernatants were collected after 24 h and 48 h transfection. RBD expression was detected using an anti-RBD monoclonal antibody *via* Western blotting (WB).

### Western Blotting

After performing electrophoresis using 4-12% NuPAGE Bis-Tris Gel (NP0321BOX, Invitrogen, USA), proteins were transferred onto membranes and blocked with 5% BSA for 30 min. Membranes were incubated with the primary antibody for 1 h, washed three times with PBST (phosphate buffered saline with Tween 20), incubated with the secondary antibody for 1 h, washed 3 times with PBST, and then developed using an ECL luminescent solution. Images were generated using X-ray film.

### Packaging of the Recombinant Adeno-Associated Virus

HEK293 cells were seeded onto a 10-cm culture dish at a density of 40,000 cells/cm^2^, and the cells in one of the dishes were counted after 24 h of culture. The total amount of plasmids to be transfected was calculated based on 1 μg of plasmid having the ability to transfect one million cells, pAAV-RC9 dosage, recombinant eukaryotic expression of plasmid, and pAAV-Help. They were combined at a mass ratio of 1:1:1. The three plasmids were added into 0.5 mL of a high-sugar DMEM medium and vortexed for 3-4 s. A fixed amount of FectoVIR-AAV was added. The solution was vortexed for 3-4 s, and the amount of FectoVIR-AAV and transfected plasmids combined at a 1 μL:1 μg ratio. After incubation for 30 min at room temperature, the mixture was added onto the HEK293 cells, and incubated at 37°C with 5% CO_2_ conditions for 72 h. At the end of incubation, the packaged cell culture supernatant and cells were collected.

### Preliminary Purification of Recombinant Adeno-Associated Virus

The collected cells were resuspended in 1 mL of PBS, and frozen. If necessary, cells had a maximum of 3 thawing cycles between liquid nitrogen and room temperature. After each thawing step, the cells were hand-shaken vigorously for 2 min to break the cells and release the virus. The lysed cells were centrifuged at 10,000 g for 10 min at 4°C. The supernatant was collected and combined with the cell culture supernatant from the initial harvest containing recombinant AAV virus. Benzonase was added at a concentration of 50 U/mL to the above solution to remove the residual DNA. The solution was incubated at 37°C for 30 min, and inverted every 10 min to ensure that benzonase was evenly mixed. Then, the solution was centrifuged at 10000 g for 20 min at 4 °C. The supernatant was filtered with a 0.45 μm filter and the filtrate was transferred to a new tube. The supernatant was transferred to an ultracentrifuge tube and density gradient centrifugation (iodixanol) was performed for 100 min at 300,000 g and 12°C. The liquid containing the virus (transparent liquid) was collected, desalted, and concentrated.

### Titer Determination of Recombinant Adeno-Associated Virus

Serial diluted RBD plasmid in the range of 10^9^ to 10^4^ genome copies was used as the standard to quantify the concentration of recombinant adeno-associated virus. Two μL of AAV vector samples were incubated at 37°C in 99 μL of DNase digestion buffer (400 mM Tris-HCl, pH 8.0, 100 mM MgSO_4_, 10 mM CaCl_2_, and 10 units DNase I) for 30 min, and treated by adding 99 μL of stop solution (20 mM EDTA, pH 8.0) following an incubation at 65°C for 10 min. Then, 20 μL of protease K digestion buffer (100 mM Tris, pH 8.0, 2 mM EDTA, 1% SDS, and 2 mg/mL proteinase K) was added and incubated at 56 °C for 90 min followed by an incubation at 95°C for 15 min. After treatment, the AAV vector samples were diluted at1000-fold in ddH_2_O. A qPCR reaction system (PowerUp SYBR Green Master Mix, Thermo fisher scientific) with a total volume of 20 μL was set up in duplicate for each sample in accordance with the manufacturer’s instructions. Specifically, each reaction mixture contained 5 μL of the diluted AAV vector sample and 0.5 μL of each of the designed primer pairs, forward primer TGGGACTTTCCTACTTGGCA, and reverse primer CCACGCCCATTGATGTACTG. The qPCR program was 50°C for 2 min and 95 °C for 2 min, followed by 40 cycles at 95°C for 15 sec and 60°C for 30 sec. The AAV genome titers were calculated according to a standard curve using the 2^^-ΔΔCT^ method.

### Purity Determination of Recombinant Adeno-Associated Virus

In a 200- μL Eppendorf tube, the standard rAAV9 vector was diluted to obtain solutions with 1×10^10^ and 2×10^10^ copies. For AAV vector samples, 1 μL, 2 μL, or 4 μL of each sample was added to one tube. ddH_2_O was added to each tube and the final volume was 15 μL. Then, 3 μL of 6× loading buffer was added to each tube and the tubes were incubated at 95°C for 10 min. The capsid proteins were assessed by performing 10% SDS-PAGE at 120 V for 90 min. Silver staining was performed at room temperature according to the manufacturer’s instructions (Pierce, OJ190653) The AAV titers were semi-quantified using the standards.

### Verifying the Expression of Recombinant Adeno-Associated Virus

HeLa cells were seeded onto a 24-well plate at a density of 40,000-50,000 cells/well. After a 24 h of inoculation, the purified recombinant adeno-associated virus was diluted to 5×10^6^ vg/μL (the titer was determined using the AAVpro Titration Kit Ver.2) and 10 μL per well added to cells (10 μL per well) in the 24-well plate. The cells were incubated at 37 °C for 48 h with 5% CO_2_. At the end, cell lysates and cell culture supernatants were collected and used for the determination of the expression of RBD by Western blotting.

### Immunization of Mice

Female Balb/c mice (6-8 weeks) were randomly divided into groups of 10 animals per group. Each animal was immunized with 10^11^ genome copies of recombinant adeno-associated virus in 50 μL of PBS. For intramuscular injection, the 50 μL samples were divided and injected into each rear leg at two sites. For nasal drip immunization, a total of 50 μL were applied to the both of their nostrils. A negative control was immunized with PBS by either intramuscular injection or nasal drops. The neutralizing antibody titer in the serum was detected before immunization and at 4 and 8 weeks after immunization.

### Enzyme-Linked Immunosorbent Assay (ELISA)

The sera of immunized mice were collected by tail bleeding the day before immunization at different time points, and were analyzed for the presence of anti-RBD antibodies with ELISA. The sera of non-immune mice served as the negative control. 96-well plate were coated with RBD protein to serve as the antigen. Serial diluted serum samples were then added to each well and the plate was incubated at 4 °C overnight. After washing with PBS, secondary antibodies labeled with streptavidin–horseradish peroxidase (HRP) were added to each well. After washing and color development, the absorbance was read at 492 nm. The end-point titers of the antibodies were determined as the reciprocal of the highest dilution, giving an optical density twice that of the non-immune serum. The geometric mean value of end-point titers (GMT) of antibodies in each group was calculated.

### The Detection of Neutralizing Antibody

The serum to be evaluated (Nie et al., 2020a; Nie et al., 2020b) was treated in a water bath at 56°C for 30 min to inactivate complement compoents, centrifuged at 6000 g for 3 min, and the supernatant was collected. The supernatant was serially diluted and added to 96-well plates. Then, 650 TCID 50/mL of novel corona pseudovirus was added to each well and the plate was incubated at 37 °C and 5% CO_2_ for 1 h. Then, 2×10^4^ Huh-7 cells were added to each well and cultured at 37°C and 5% CO_2_ for 24 h. Next, 75 μL of liquid was removed from each well and dispensed to a new 96-well plate,75 μL of luciferase detection reagent was added to each well. The 96-well plate was placed on a chemiluminescence detector after a 3 min incubation at room temperature and the neutralization inhibition rate was calculated using the following equation: Inhibition rate = [1 - (mean luminous intensity of the sample group - mean value of blank control)/(mean luminous intensity of the negative group - mean value of blank control)] × 100%.

Based on the results of the neutralization inhibition rate, the neutralizing antibody EC_50_ was calculated using the Reed-Muench method. If the EC_50_ value was less than 30, it was determined to be 15.

### Enzyme-Linked Immunospot Assay (ELISPOT)

Splenocytes from immunized mice were analyzed for cytokine production using an ELISPOT kit (DAKEWE, China) following the manufacturer’s instructions. Specifically, the levels of interferon (IFN)-γ, interleukin (IL)-2, IL-4, and IL-10 were determined to evaluate the immune responses *in vivo*. In brief, splenocytes were placed on 96-well filtration plates pre-coated with capturing Abs at 10^5^ cells/well and then stimulated with an overlapping spike glycoprotein peptide pool. Splenocytes stimulated with PMA+ Ionomycin served as a positive control, and RPMI 1640 medium alone served as a negative control. After incubation with biotinylated detection Abs followed by streptavidin-HRP, spots at the sites of cytokine secretion were developed by adding a 3-amino-9-ethylcarbazole substrate and were counted automatically by an ELISPOT reader system (Mabtech, Switzerland).

## Results

### Construction of Recombinant Eukaryotic Expression Plasmid

**Figure 2:**
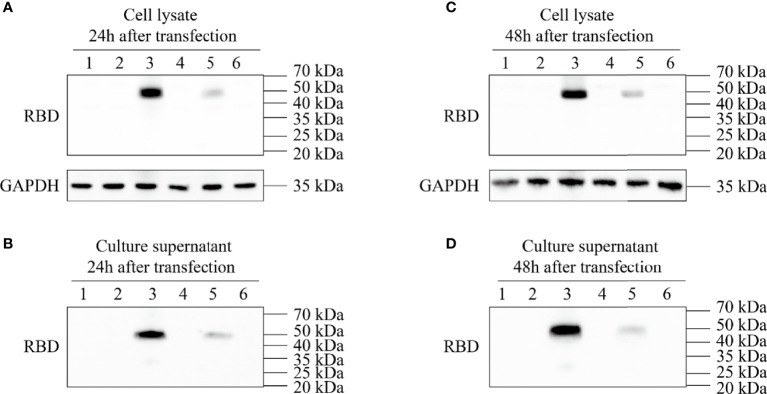
Expression identification of recombinant plasmid pAAV-RBD. HEK293 cells were transfected with the four different plasmids and the RBD expressions were detected in the cell lysates or supernatants. 1: Negative control; 2: Clone 1; 3: Clone 2; 4: Clone 3; 5: Clone 4; 6: EGFP plasmid. **(A)** Expression of RBD in the cell lysate 24 hours post-transfection. **(B)** Expression of RBD in the culture supernatant 24 hours post-transfection. **(C)** Expression of RBD in the cell lysate 48 hours post-transfection. **(D)** Expression of RBD in the culture supernatant 48 hours post-transfection.

After the synthesized target genes (**Figure 1**) were ligated into the ssAAV-MCS (single chain AAV expression plasmid) vector or scAAV-MCS (self-complementary AAV expression plasmid) vector at the BamH I and Sal I digestion sites, the recombinant expression plasmid with the correct sequence was transfected into 293T cells. The cell lysate and culture medium supernatant were collected at 24 and 48 h after transfection, respectively, and the expression of RBD was detected *via* an RBD-specific antibody by WB. The four recombinant expression plasmids constructed in the experiment were as follows: clone no. 1: scAAV-RBD (tPA signal peptide), clone no. 2: ssAAV-RBD (tPA signal peptide); clone no. 3: scAAV-RBD (Glu signal peptide); and clone no. 4: ssAAV-RBD (Glu signal peptide). The results are shown in **Figure 2**. The expression of recombinant RBD was detected in the cell lysate and cell culture supernatant 24 h after the transfection of the recombinant expression plasmid (clone no. 2 and clone no. 4). Meanwhile, the expression of recombinant RBD was also detected in the cell culture supernatant 48 h after transfection, and a little higher expression levels of recombinant RBD were observed in the cell culture supernatant at that time. The molecular weight was approximately 50 Kda, which is larger than the expected 27 KDa due to glycosylation.

### The Expression and Identification of Recombinant AAV9-RBD Virus

**Figure 3:**
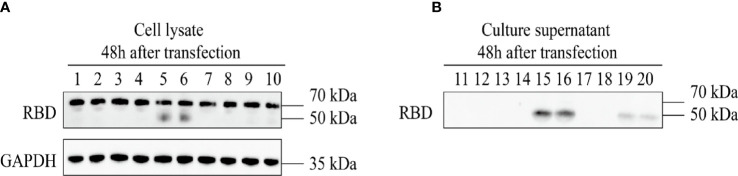
Expression identification of recombinant AAV9-RBD. The four different AAV vectors were packaged in duplicate and used to infect HeLa cells. The expression of RBD in the cell lysates and supernatants were detected at 48 hours after infection. 1,2: AAV9-EGFP; 3,4: AAV vectors from clone 1; 5,6: AAV vectors from clone 2; 7,8: AAV vectors from clone 3; 9,10: AAV vectors from clone 4. **(A)** Expression of RBD in the cell lysate at 48 hours after AAV vector infection. **(B)** Expression of RBD in the culture supernatant at 48 hours after AAV vector infection.

The 293T cells were co-transfected with four groups of three plasmids, namely, pAAV-RC9, pAAV-Help, and one of the four constructed recombinant plasmids. The packaged recombinant AAV9 virus was collected 72 h later. The sequence numbers of the four recombinant viruses corresponded to the sequence numbers of the four recombinant expression plasmids. The plasmids were used to infect HeLa cells that were assessed by Western Blot. After 48 h, the expression of recombinant RBD was detected in the cell lysate and cell culture supernatant of the recombinant AAV9-RBD virus infection group packaged with clone no. 2 (virus no. 2, single-chain AAV9-RBD; the signal peptide was tPA signal peptide). Moreover, the concentration of the target protein in the cell culture supernatant was much higher than that in the cell lysate (**Figure 3**). While other viruses were either not expressed (virus no. 1, self-complementary AAV9-RBD, the signal peptide was tPA signal peptide; virus no. 3, self-complementary AAV9-RBD, the signal peptide was Glu signal peptide) or low in expression in cell culture supernatant (virus no. 4, single-chain AAV9-RBD; the signal peptide was Glu signal peptide). Virus no. 2 was used in subsequent trials.

### Packaging, Purification, and Titer Determination of Recombinant AAV9-RBD Virus

**Figure 4:**
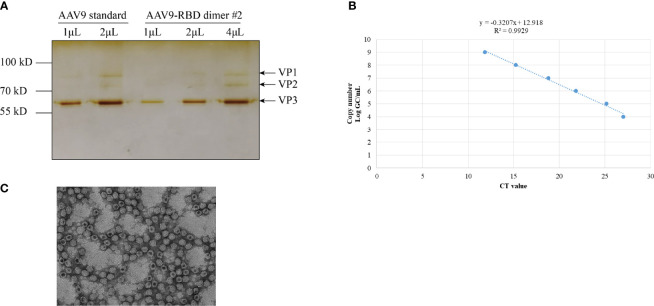
AAV vector quantification by sliver staining and Q-PCR. The AAV vector was characterized by Silver staining **(A)**, Q-PCR **(B)** and electron microscopy **(C)**.

After the recombinant AAV9-RBD virus was purified, the level of purity was determined by silver nitrate staining (**Figure 4A**). Three major bands representing VP1, VP2, and VP3 were detected in both standard and AAV9-RBD viruses. No bands were observed for the recombinant AAV9-RBD virus, indicating that the purity level were sufficient for animal experiments. Approximately 10^13^ recombinant AAV9-RBD viral particles/mL were detected. After Q-PCR, 2 mL of the recombinant AAV9-RBD virus solution with a genome titer of approximately 2.4×10^13^ GC/mL (the recombinant AAV9-RBD virus packaged by clone no. 2) was detected (**Figure 4B**).

### Humoral Immune Responses in AAV9-RBD Virus Immunized Mice

**Figure 5:**
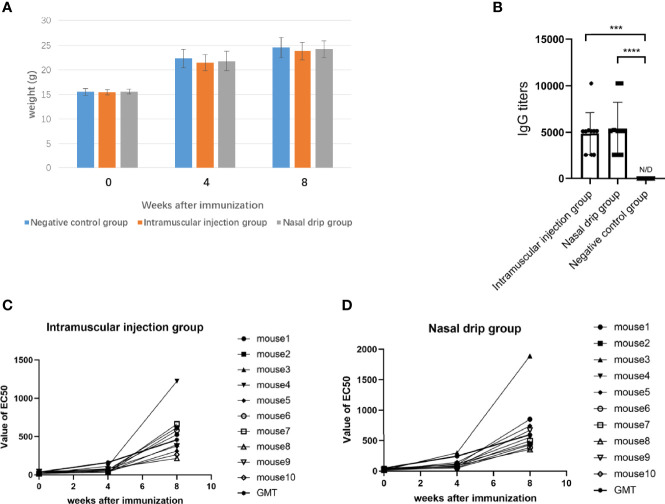
The mice were immunized with recombinant AAV9-RBD *via* intramuscular injection and nasal drip administration. Weight changes of mice before and after immunization **(A)**. RBD-specific antibody responses in sera of immunized mice 8 weeks after immunization. Endpoint titers of IgG determined by ELISA. The reciprocals of titers were represented as the mean ± SD. n = 10. ***p＜ 0.0003; ****p＜0.0001 **(B)**. The levels of the neutralizing antibody in each mouse group were determined before immunization, and 4 and 8 weeks after immunization *via* intramuscular injection **(C)** and intranasal administration **(D)**.

After 8 weeks of administration, all mice survived and weighed (**Figure 5A**). The humoral immune response, including specific IgG and neutralizing antibodies were determined after two immunizations. The results were shown in **Figure 5**. The AAV9-RBD virus elicited the production of specific an IgG antibody against RBD with a GMT of 1:4873 (intramuscular injection) and 1:5385 (nasal drip), respectively, representing a significant difference (*p＜0.0003*). The EC_50_ values of the neutralizing antibody titers for each mouse group were mostly less than 30 before immunization. The neutralizing antibody was generated 4 and 8 weeks after mice were immunized. In the intramuscular injection group, the EC_50_ value of the neutralizing antibody was 24.0 ± 12.0 (n=10) before immunization, 61.6 ± 25.5 (n=10) at the 4^th^ week, and 517.7 ± 292.1 (n=10) at the 8^th^ week, representing a 2.6 and 21.6-fold increase, respectively. In the nasal drip group, the EC_50_ value of neutralizing antibody before immunization was 24.9 ± 13.3 (n=10), 113.2 ± 70.6 (n=10) at the 4^th^ week, and 682.8 ± 454.0 (n=10) at the 8^th^ week; thus, an 4.5-and 27.4-fold increase, respectively, was observed. In short, the results showed that vaccination with the AAV9-RBD virus expressing RBD antigens induced both humoral and RBD-specific strong antibody responses in mice.

### Cytokine Production in AAV Vectored COVID-19 Vaccine Immunized Mice

**Figure 6:**
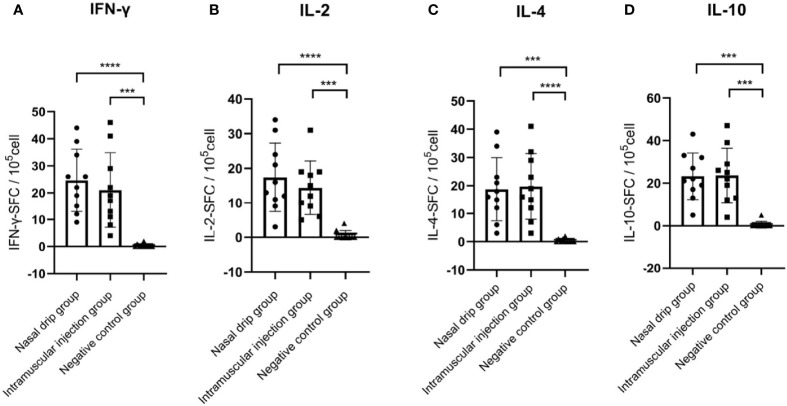
Levels of splenocyte secreted cytokines in immunized BALB/c mice at 8 weeks after immunization detected by ELISPOT. **(A)** IFN-γ, **(B)** IL-2, **(C)** IL-4, and **(D)** IL-10 levels. The numbers of cytokinepositive cells were shown as the mean counts of spot-forming unit (SFU) ± SD per 1×10^5^ splenocytes. n = 10. Results were analyzed by the t test. ^***^p＜ 0.0003; ^****^p＜0.0001.

To determine the cellular immune response, levels of splenocyte-derived cytokines were determined by ELISPOT at 8 weeks after immunization. Significant increases in the levels of IFN-γ, IL-2, IL-4, and IL-10 were detected in the nasal drip group and intramuscular injection group compared to those of the negative control (p< 0.01, **Figure 6**). These findings from intracellular cytokine staining and cytokine profiling suggested a AAV9-RBD-induced T-helper-2 and T-helper-1 cell dominant response after both intramuscular injection and nasal drip administration.

## Discussion

Due to its high gene delivery efficiency, low immunogenicity, lack of pathogenicity, and excellent safety profile, the adeno-associated virus is a preferred vector for gene therapy (Kotterman and Schaffer, 2014; Naso et al., 2017). After the recombinant adeno-associated virus infects cells, it releases its viral DNA into the cell, synthesizes the complementary chain after entering the nucleus, and connects the head and tail into a ring, to form a double-stranded chain, which can exist in the free form in mammalian cells for a long time without being degraded as exogenous DNA (Carter, 2004; Penaud-Budloo et al., 2008), thus achieving the long-term and stable expression of exogenous genes. At present, there are 12 AVV serotypes (AAV1 to AAV 12) and more than 130 mutants of AAV in existence (Daya and Berns, 2008). Different AAV serotypes have different spatial structures, capsid protein sequences and tissue specificities. Hence, the cell surface receptors that recognize and bind to the AAV are also different, which results in different cell types being transfected by different serotypes of AAV with varying infection efficiencies (Mohan et al., 2003; Burger et al., 2004; Evans et al., 2006).

We selected the AAV9 virus, which had a relatively wide tissue tropism, for constructing the SARS-CoV-2 vaccine, as it would enable us provide a low dose of viral inoculum to achieve a desirable immunity and safety. In 2019, the FDA approved the recombinant AAV9 drug Zolgensma, produced by Novartis, for the treatment of spinal muscular atrophy in children, which demonstrated the safety of this vector. The viral shell of recombinant AAV9 (rAAV9) can be used as an adjuvant to promote the induction of the neutralizing antibody against the recombinant RBD protein. In addition, rAAV9 may infect mammals *via* a nasal spray (Demminger et al., 2020). Nasal delivery of rAAV9 vaccine is not only convenient, but also provide protection to the nasal and lung mucosa.

To further improve antigen expression and reduce the dosage of the recombinant virus, we introduced two copies of RBD with a flexible linker region in the middle to ensure that the expressed RBD has the correct conformation. To achieve the secretory expression of the recombinant RBD protein, we included the signal peptide of the human tissue plasminogen activator (tPA) at the N-terminus of the recombinant RBD protein. The signal peptide was hydrolyzed after the recombinant protein was secreted outside the cells (Choo and Ranganathan, 2008). To find a suitable signal peptide, we also compared the effect of the glutamate (Glu) signal peptide on recombinant protein secretion (clone no. 4 and recombinant virus 4). As demonstrated in **Figures 2**, **4** that the tPA signal peptide was stronger than the Glu signal peptide in promoting secretion of the recombinant RBD during the expression of eukaryotic plasmids. Glu signal peptide’s effect was much weaker than that observed with the tPA signal peptide during the recombinant viral infection. Therefore, we used the tPA signal peptide for the construction of rAAV-RBD.

At week 8 after immunization with a single dose of AAV-RBD, mice in the intramuscular injection and nasal drip groups had high levels of anti-pseudovirus neutralizing antibodies with EC_50_ values of 517.7 and 682.8, respectively. These antibody levels exceeded those shown to have protective effects against SARS-CoV-2 infection (Robbiani et al., 2020; Khoury et al., 2021). We speculate that the neutralizing antibody levels in these mice may continue to rise over time. The results of our experiment suggested that the AAV9-RBD virus vaccine when administered by intramuscular injection or a nasal spray is a promising candidate worthy of further development.

Cellular and humoral immune responses are considered to be equally important for the prevention against SARS-CoV-2 as a safe and efficient vaccine. In this study, IFN-γ, IL-2, IL-4, and IL-10 were all markedly increased in the nasal drip and intramuscular injection groups. The Th1 response is mediated by Th1 helper cells with the production of IFN-γ and IL-2, which are associated with the cellular immune response, while the Th2 response is closely related with the production of IL-4 and IL-10, which boosts the humoral response. These findings from the intracellular cytokine staining and cytokine profiling suggested that the AAV9-RBD virus induced balanced cellular and humoral responses after both intramuscular injection and nasal drip administration.

The blood samples were taken from the retro-orbital sinus in mice *via* capillary tubes at different time points. We did not measure the neutralization antibodies for AAV9 because the blood samples at different time points were used for the calculation of RBD antibody titers, the main purpose of this study, and there was not enough for quantification of AAV9 antibody titers. Injection of AAV9 vector either in muscle or intranasal can elicit neutralization antibodies in mice, which is well documented (Murrey et al., 2014).

There are also other AAV based vaccines for COVID-19, like AAVhu68-ACE2 (Sims et al., 2021) and AAVrh32.33-S (Zabaleta et al., 2021). Compared with them, the pros for AAV9-RBD are (1) two linked RBDs are more efficient antigens to elicit immune responses than single S protein or S1 protein, which has been showed by recombinant protein vaccine study. (2) AAV9 can infect both muscle and airway epithelia cells efficiently to overexpress RBD to elicit immune response. While, the cons for AAV9-RBD are (1) in the absence of AAV9 neutralizing antibody inhibitors, the vector may not be used for 20-40% patients that have the AAV9 neutralization antibodies while AAVrh32.33 may be used for more patient due to less neutralization antibody in population; (2) the overexpression of S or S1 protein may elicit more immune protection due to more antigens.

The recombinant AAV9-RBD virus vaccine induced sustained expression of RBD in infected host cells over a long period, and stimulated the immune system to produce long-lasting antibody and cellular immune responses. Therefore, the AAV9-RBD virus vaccine has the potential of offering long-term protection against SARS-CoV-2. The vaccine may be used to immunize naïve humans or as a booster vaccine for those who have previously been immunized but with a waning immunity.

## Data Availability Statement

The raw data supporting the conclusions of this article will be made available by the authors, without undue reservation.

## Ethics Statement

The animal study was reviewed and approved by Institutional Animal Care and Use Committee of Beijing Institute of Biotechnology.

## Author Contributions

Conceived and designed the experiments: YZ and YHL. Performed the experiments: XQ and SL. Contributed reagents/materials/analysis tools: BD and YL. Wrote the paper: XQ and XL. Participated in the experiments: YD, LL, HB, XS, EF, LZ, and YA. Guided the experiments: DP and YG. Data analysis: FH and LY. Guided the writing and revised the manuscript: SL and YZ. All authors contributed to the article and approved the submitted version.

## Conflict of Interest

The authors declare that the research was conducted in the absence of any commercial or financial relationships that could be construed as a potential conflict of interest.

## Publisher’s Note

All claims expressed in this article are solely those of the authors and do not necessarily represent those of their affiliated organizations, or those of the publisher, the editors and the reviewers. Any product that may be evaluated in this article, or claim that may be made by its manufacturer, is not guaranteed or endorsed by the publisher.
